# An improved lightweight object detection algorithm for YOLOv5

**DOI:** 10.7717/peerj-cs.1830

**Published:** 2024-01-30

**Authors:** Hao Luo, Jiangshu Wei, Yuchao Wang, Jinrong Chen, Wujie Li

**Affiliations:** 1 College of Information Engineering, Sichuan Agricultural University, Ya’an, Sichuan, China; 2 College of Mechanical and Electrical Engineering, Sichuan Agricultural University, Ya’an, Sichuan, China

**Keywords:** Lightweight object detection, Deep learning, YOLOv5, Ghost module, Attention mechanisms

## Abstract

Object detection based on deep learning has made great progress in the past decade and has been widely used in various fields of daily life. Model lightweighting is the core of deploying target detection models on mobile or edge devices. Lightweight models have fewer parameters and lower computational costs, but are often accompanied by lower detection accuracy. Based on YOLOv5s, this article proposes an improved lightweight target detection model, which can achieve higher detection accuracy with smaller parameters. Firstly, utilizing the lightweight feature of the Ghost module, we integrated it into the C3 structure and replaced some of the C3 modules after the upsample layer on the neck network, thereby reducing the number of model parameters and expediting the model’s inference process. Secondly, the coordinate attention (CA) mechanism was added to the neck to enhance the model’s ability to pay attention to relevant information and improved detection accuracy. Finally, a more efficient Simplified Spatial Pyramid Pooling—Fast (SimSPPF) module was designed to enhance the stability of the model and shorten the training time of the model. In order to verify the effectiveness of the improved model, experiments were conducted using three datasets with different features. Experimental results show that the number of parameters of our model is significantly reduced by 28% compared with the original model, and mean average precision (mAP) is increased by 3.1%, 1.1% and 1.8% respectively. The model also performs better in terms of accuracy compared to existing lightweight state-of-the-art models. On three datasets with different features, mAP of the proposed model achieved 87.2%, 77.8% and 92.3%, which is better than YOLOv7tiny (81.4%, 77.7%, 90.3%), YOLOv8n (84.7%, 77.7%, 90.6%) and other advanced models. When achieving the decreased number of parameters, the improved model can successfully increase mAP, providing great reference for deploying the model on mobile or edge devices.

## Introduction

In recent years, with the development of convolutional neural networks, the computer field has ushered in great changes, and various intelligentization has entered the public’s vision, providing great convenience for people’s lives. Ding et al. (2020) proposed a system based on fraud detection algorithm, which can effectively detect fraudulent trips without taxi meters; Liao et al. (2021) proposed a new multi-task learning model for fake news detection, enhancing the identification performance, particularly for short fake news. Wu et al. (2018) propose a hybrid semisupervised learning model for spam detection based on hybrid PU-learning, which successfully finds and validates hidden spammers. Target detection, one of the core tasks in the field of computer vision, is even more greatly changed. The target detection task is to find all objects of interest in the image and determine their category and location (Arifando, Eto & Wada, 2023; Appe, Arulselvi & Balaji, 2023). Unlike traditional object detection algorithms, deep learning-based detection methods can learn deeper semantic features, enhancing the model’s expression ability and improving recognition accuracy.

Currently, object detection algorithms based on deep learning are divided into two categories: two-stage algorithms and one-stage algorithms. Two-stage target detection algorithm first generates regions and then classifies and regresses samples through a convolutional neural network. The one-stage object detection algorithm directly extracts features from the network to predict object category and location. Standard one-stage object detection algorithms include YOLO series algorithms (Wang, Yeh & Liao, 2021; Bochkovskiy, Wang & Liao, 2020; Wang, Bochkovskiy & Liao, 2023), RetinaNet (Lin et al., 2017).

The two-stage object detection algorithm utilizes a sizable network model. While it achieves high detection accuracy, it necessitates the generation of numerous candidate regions, leading to slower detection speed and subpar real-time performance. Consequently, it is not conducive to deployment on mobile devices. In contrast, the one-stage object detection algorithm boasts rapid detection speed and strong real-time performance, as it operates without the need for candidate regions. Tang et al. (2023) improved the YOLOv7 model for detecting plums in natural environment by adding the attention mechanism and modifying the upsampling function, which improved mAP by 2.03%; Muhammad et al. (2023) Using the C3 and FPN + PAN structures and attention mechanism, the original YOLOv5 model has been enhanced in the backbone and neck section to achieve high identification rates. Liu et al. (2022) further expanded the model to 3 billion parameters by leveraging the Swin Transformer (Liu et al., 2021) architecture, allowing it to accommodate training images with resolutions up to 1,536 × 1,536. This extension resulted in an impressive mAP of 63.4 on the COCO dataset. Zheng et al. (2023) proposed a lightweight YOLOv5 model to detect ships, and the detection accuracy increased by 3.4%. Lou et al. (2023) proposed a small-size object detection algorithm based on YOLOv8 model for special scenes, which improved the detection accuracy of small-size objects. Yang et al. (2023) proposed a method for real-time detection of forest fires based on YOLOv5 network, the mAP increased from 72.6% to 89.4%, comprising an accuracy improvement of 16.8%. Zhang et al. (2022) proposed an improved model based on YOLOv5 and used it to detect orchard pests, the results show that the mAP of the proposed method increases by 1.5% compared to the original YOLOv5. In summary, the YOLOv5 model boasts rapid training and inference speeds, making it well-suited for a diverse range of applications.

The above methods can improve the detection accuracy of the model well, but the number of model parameters and the required computing resources are too large, and it is not friendly to the edge devices with limited memory and computing resources. In order to better deploy the model on mobile or edge devices with limited computing power and memory, there is a need to refine the model for lightweight performance. However, the reduction of the number of model parameters is often accompanied by the reduction of the detection accuracy. To address this problem, we proposed a lightweight object detection algorithm based on YOLOv5s. Firstly, by virtue of the lightweight characteristics of the Ghost module, it was added to the C3 structure. At the same time, C3 modules with larger output channels were replaced, and other C3 modules with smaller output channels were retained. Secondly, in order to solve the problem of reduced feature extraction ability of the model caused by the reduction of parameters, we focused on more critical information by adding the CA mechanism (Hou, Zhou & Feng, 2021), thereby improving the detection performance of the model; finally, to reduce the effect of the attention mechanism reducing the training speed, we replaced the sigmoid linear unit (SiLU) function in the SPPF module with the rectified linear unit (ReLU) function, which improved the training speed of the model and enhances the stability of the model.

To conclude, the main contributions are as follows:

1) Different from the method of replacing backbone as a whole or replacing all modules to lightweight modules, only some C3 modules were replaced with a large number of output channels. This trick allows for model lightweighting without significantly increasing the network depth through the addition of too many Ghost modules and it does not affect the ability of model backbone network to extract features.
2) Unlike other attention mechanisms that only focus on channel or spatial information, CA mechanism can focus on key information on channels and space at the same time, which will greatly improve the model’s ability to extract features.
3) The proposed model has significant performance improvements on datasets with different characteristics, and the generalization ability of the proposed method was studied on the Pascal VOC dataset. Compared with other lightweight SOTA models, such as YOLOv7tiny and YOLOv8n, it still has higher detection performance.
4) When achieving the decreased number of parameters, the improved model can successfully increase mAP, providing great reference for deploying the model on mobile or edge devices.

The remainder of this article is organized as follows. In the Materials and Methods section, we will introduce the improved model, experimental settings, and evaluation indicators. The Experiment Results and Discussion section introduces the experimental results of original model, improved model and other state-of-the-art models on three different feature datasets. The Conclusions section introduces the relevant contributions of this article and prospects for future work.

## Materials and Methods

### YOLOv5 network

The YOLOv5 detection algorithm was introduced by Glenn Jocher in 2020 (Jocher et al., 2020), and it demonstrates significant advantages in rapid model deployment. According to YOLOv5 the model can be categorized into several versions, namely YOLOv5n, YOLOv5s, YOLOv5m, YOLOv5l, and YOLOv5x, which vary in terms of network depth and width, resulting in different parameter numbers and computational complexity levels. In order to achieve high detection accuracy while remaining suitable for deployment on mobile devices, the YOLOv5s model has been chosen as the focal point of our research. The YOLOv5 model comprises four key components: Input, Backbone, Neck, and Head.

The Input layer encompasses several aspects, including Mosaic data augmentation, adaptive anchor computation, and adaptive image scaling. Mosaic data augmentation is a technique that involves the random cropping, scaling, and merging of four images. This process enhances the background and increases the representation of smaller targets, thereby improving object detection effectiveness. The adaptive anchor training process computes the optimal anchor values based on different training sets and subsequently updates them. Adaptive image scaling ensures that input images are uniformly resized to the same dimensions, allowing them to be fed into the network without distortion.

**Figure 1:**
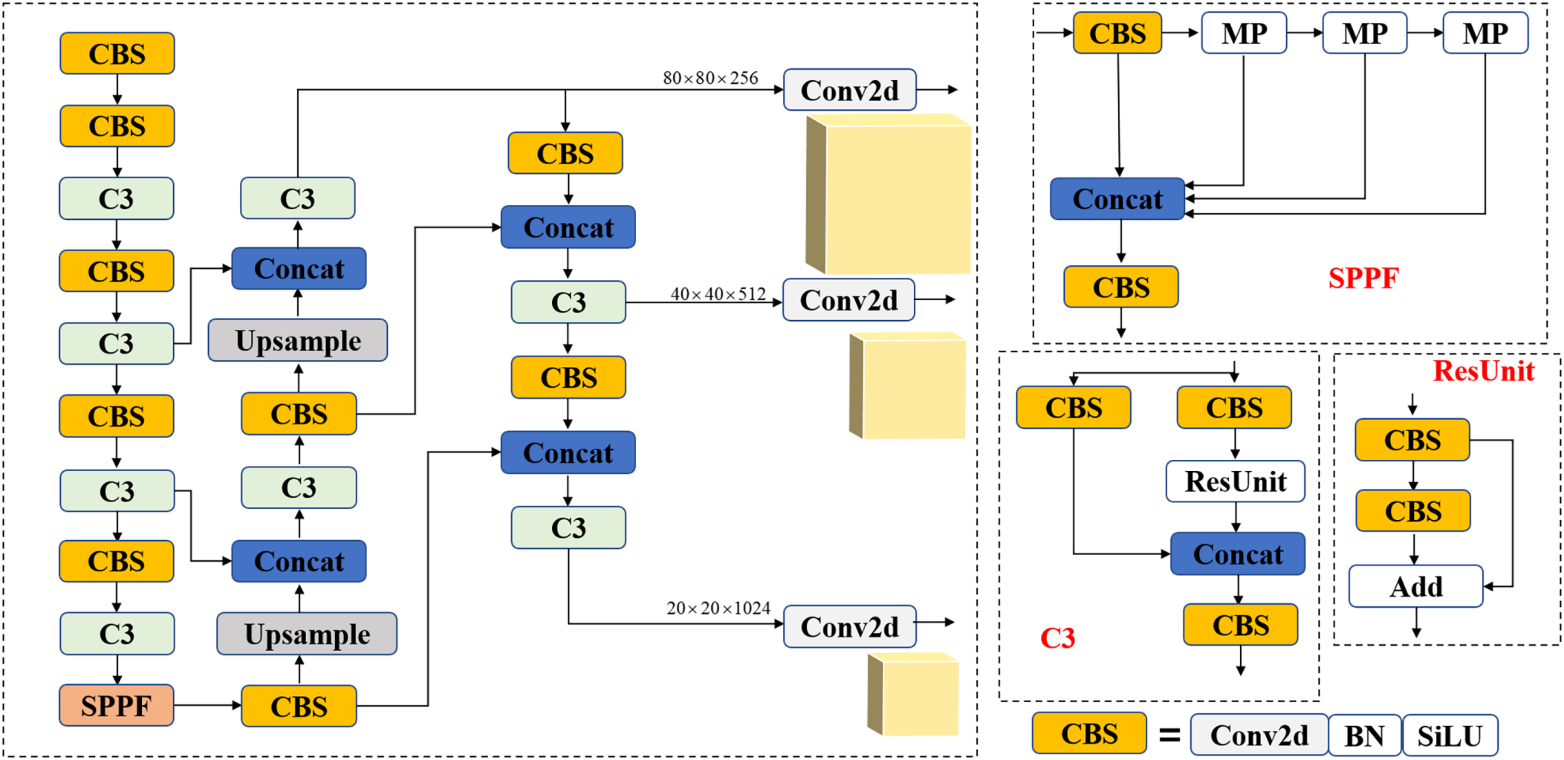
YOLOv5 model structure and module structure diagram.

Backbone is mainly composed of CBS structure, C3 structure, and SPPF structure. As shown in Fig. 1, the CBS structure is the basic convolution module of the YOLOv5 algorithm, composed of ordinary two-dimensional convolution, regularization, and the Silu Activation function. The C3 structure comprises the CBS module and the Bottleneck residual module. The SPPF structure performs the maximum pooling operation of different sizes of pooling cores on the input and performs feature fusion.

The Neck layer features a pyramid structure comprising a Feature Pyramid Network (FPN) and a Path Aggregation Network (PAN) for feature fusion. The FPN structure handles down-sampling operations to map high-level semantic information to low-level, while the PAN structure undertakes up-sampling functions to map low-level information to high-level. These two structures complement each other and significantly enhance the network’s feature fusion capabilities.

The Head layer consists of three feature detection heads, each responsible for detecting objects of different sizes within three feature maps. On the basis of the size of the feature map, three anchors are set with varying ratios of aspect for target categorization and regression prediction.

### Ghost convolutional module

**Figure 2:**
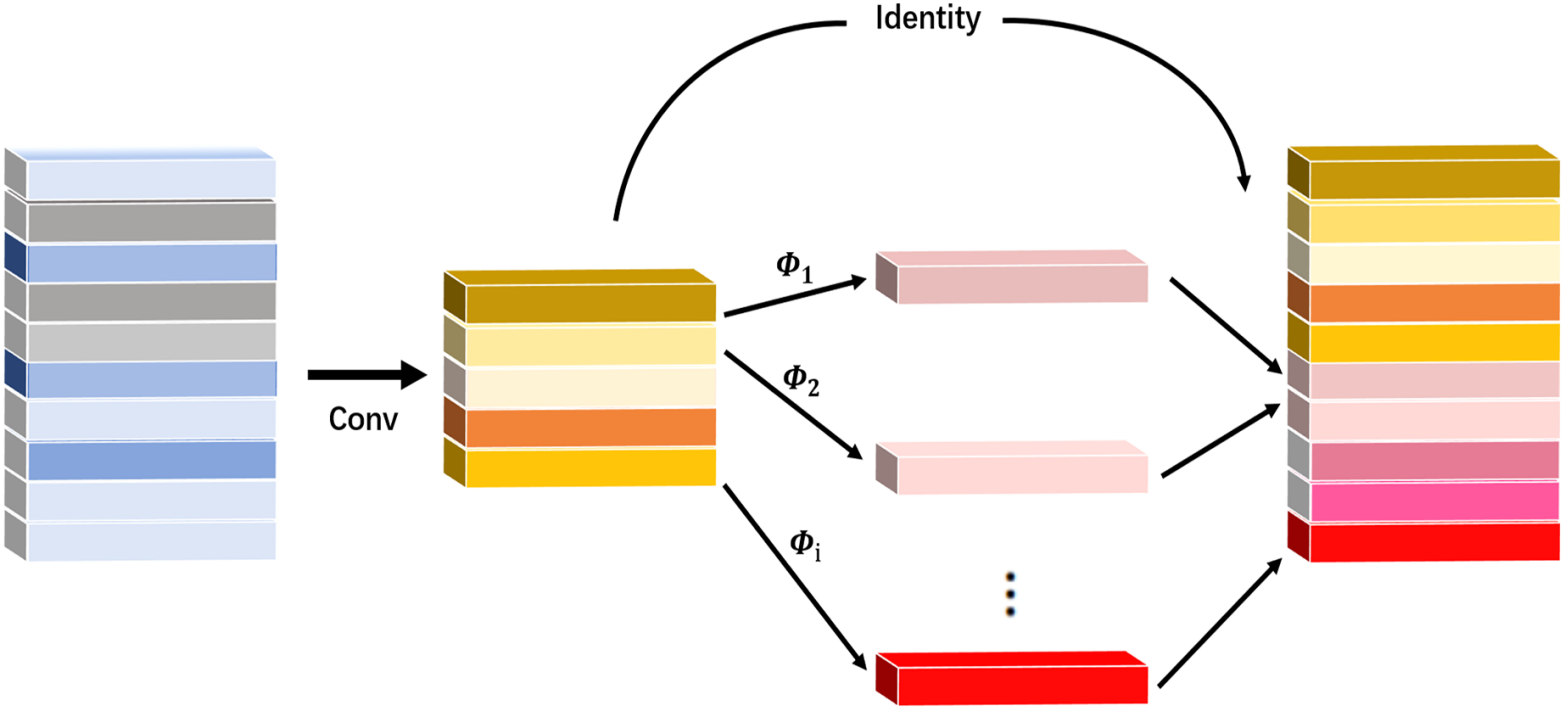
Ghost module.

Ghost Net is a new lightweight network model proposed by Han et al. (2020) on CVPR2020. This network model transforms ordinary convolutional layers into a phased convolutional calculation module. For a particular feature layer, only convolution operations are used with the module to generate a portion of the actual feature layers, and the remaining feature layers are obtained through a linear function on top of the previously developed essential feature layers, thereby extracting more feature information. Finally, the feature maps obtained from the two operations are concatenated on the specified dimensions to form a complete feature layer. The Ghost module is shown in Fig. 2.

The operation of generating feature maps for ordinary convolutions can be expressed as Eq. (1):

(1) $$Y = X*\omega + b$$

Among them $X \in {R^{h \times w \times {c_i}}}$, $h$ and $w$ are the height and width of the input data respectively, ${c_i}$ is the number of channels in input data, $b$ is the bias item, and $*\;$ indicates the convolutional operation. And $Y \in {R^{{h_o} \times {w_o} \times n}}$ indicates that when the output is $n$ channels, the feature map with a height of ${h_o}$ and a width of $\; \; {w_o}$, $\omega \in {R^{{c_i} \times k \times k \times n}}$ is the convolution filter in this layer. $k \times k$ is the kernel size of the convolution filter $\omega$. In this convolution process, the required FLOPs can be computed as $n \times {h_o} \times {w_o} \times {c_i} \times k \times k\; \;$ and since the number of filters $n$ and the number of channels ${c_i}$ are usually huge (*e.g*., 512 or 1,024), the FLOPs are also significant Ghost convolution that completes feature extraction in two steps. The first step is to obtain $m$ feature maps with fewer channels through traditional convolution. The second step is to obtain a large number of $s - 1$ feature maps through linear transformation ${\varphi _i}$. The final step is to get $n$ feature output maps by splicing the feature maps obtained in the previous two steps. Thus, in Ghost convolution, $\displaystyle{n \over s} \times {h_o} \times {w_o} \times {c_i} \times k \times k\;$ represents the FLOPs of the first step, $\left( {s - 1} \right) \times \displaystyle{n \over s} \times {h_o} \times {w_o} \times d \times d$ represents the FLOPs of the second step, and $d \times d$ represents the average kernel size of each linear transformation operation. Therefore, the FLOPs of ordinary convolution are divided by the FLOPs of Ghost convolution to obtain Eq. (2):

(2) $$r = \displaystyle{{n \times {h_o} \times {w_o} \times {c_i} \times k \times k} \over {\displaystyle{n \over s} \times {h_o} \times {w_o} \times {c_i} \times k \times k + \left( {s - 1} \right) \times \displaystyle{n \over s} \times {h_o} \times {w_o} \times d \times d}} \approx \displaystyle{{{c_i} \times s} \over {{c_i} + s - 1}} \approx s$$

**Figure 3:**
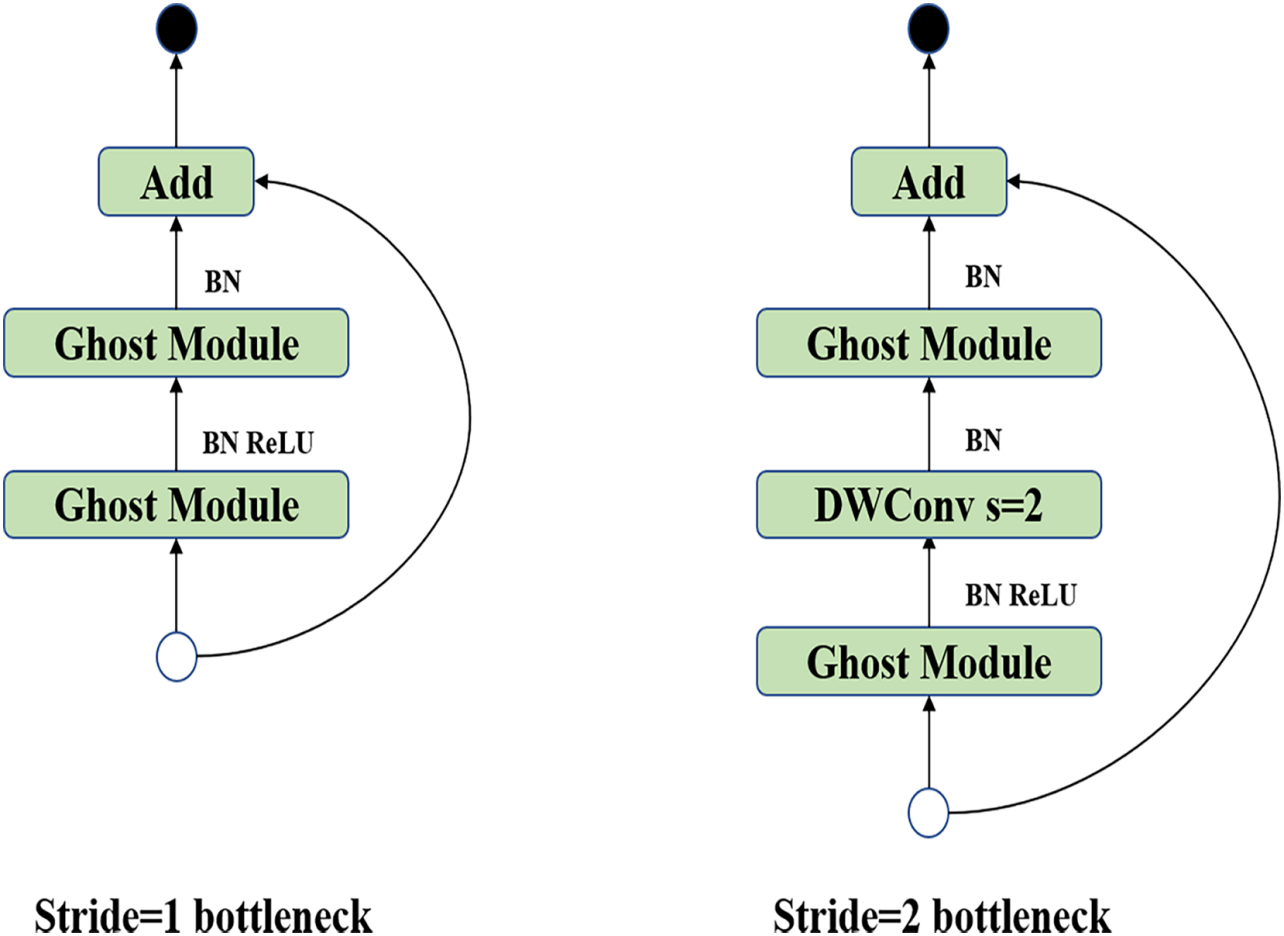
Ghost bottleneck module.

It can be seen that Ghost convolution reduces the amount of calculation by $s$ times compared with ordinary convolution, and Ghost Bottleneck is the most basic module in Ghost Net composed of Ghost modules, as shown in Fig. 3.

### Coordinate attention mechanism

**Figure 4:**
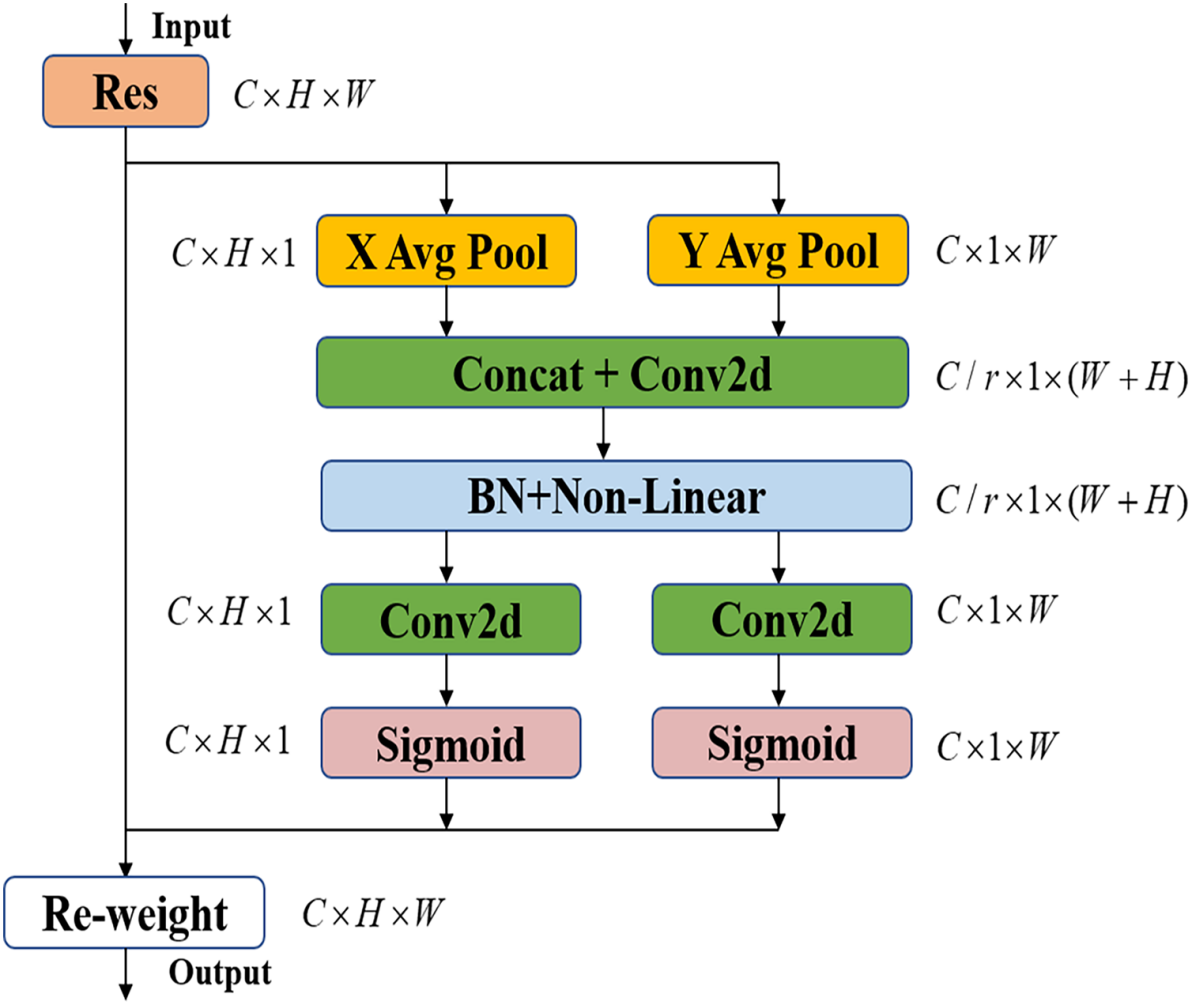
Coordinate attention mechanism.

In recent years, the Squeeze-and-Excitation (SE) (Hu et al., 2018) attention mechanism module and the Convolutional Block Attention Module (CBAM) (Woo et al., 2018) attention mechanism module have dominated all the attention mechanisms, and the SE attention mechanism only focuses on the dependencies among channels, but ignores the learning of the spatial location information of the feature map; the CBAM attention mechanism introduces a large-scale convolution kernel to extract the spatial location features of the feature map, but ignores the long-range dependencies. Through coordinate information embedding and coordinate attention generation, the CA mechanism encodes precise location information for channel relationships and long-range dependencies. As shown in Fig. 4, for a feature map with an input size of $C \times H \times W$, the pooling kernel sizes $\left( {H,1} \right)$ and $\left( {1,W} \right)$ are used to perform overall average pooling on the input feature map along the horizontal and vertical coordinates, respectively, to generate the Features ${z^h}$ and ${z^w}$. Subsequently, the information in the two spatial directions is spliced through the concatenate operation, and the $1 \times 1$ convolutional kernel is used for dimensionality reduction processing after BatchNorm processing and non-linear operation, as shown in Eq. (3):

(3) $$f = \delta ({F_1}[{z^h},{z^w}])$$

$f$ is the generated feature map with size of $C/r \times 1 \times \left( {W + H} \right)$ and $r$ is the compression factor. Then, the feature map is decomposed into tensors in the two directions, namely along the horizontal and vertical directions, and the dimension is increased through the $1 \times 1$ convolution kernel to match the number of channels. After procession through the Sigmoid activation function, the attention weights in the $h$ and $w$ directions are respectively obtained.

The CA attention mechanism takes into account not only the channel information but also the direction-related position information. It flexible and is lightweight enough to be easily integrated, making it a solution that can be applied and taken into effect instantly. As shown in Table 1, the SE module, CBAM module, and CA module are respectively integrated into the original YOLOv5s model. In the three datasets, the model utilizing the CA attention mechanism exhibits a higher mAP compared to the SE module and CBAM module. This also demonstrates that the CA attention mechanism outperforms the SE module and CBAM module in terms of performance.

**Table 1:** Comparison of various attention mechanisms in YOLOv5s (map@0.5).

Model	Helmet	Pascal VOC	Tomato
YOLOv5s	0.841	0.767	0.905
YOLOv5s (SE)	0.849	0.770	0.913
YOLOv5s (CBAM)	0.836	0.772	0.911
YOLOv5s (CA)	**0.855**	**0.775**	**0.916**

**Note:**

The best results are in bold.

### SimSPPF

**Figure 5:**
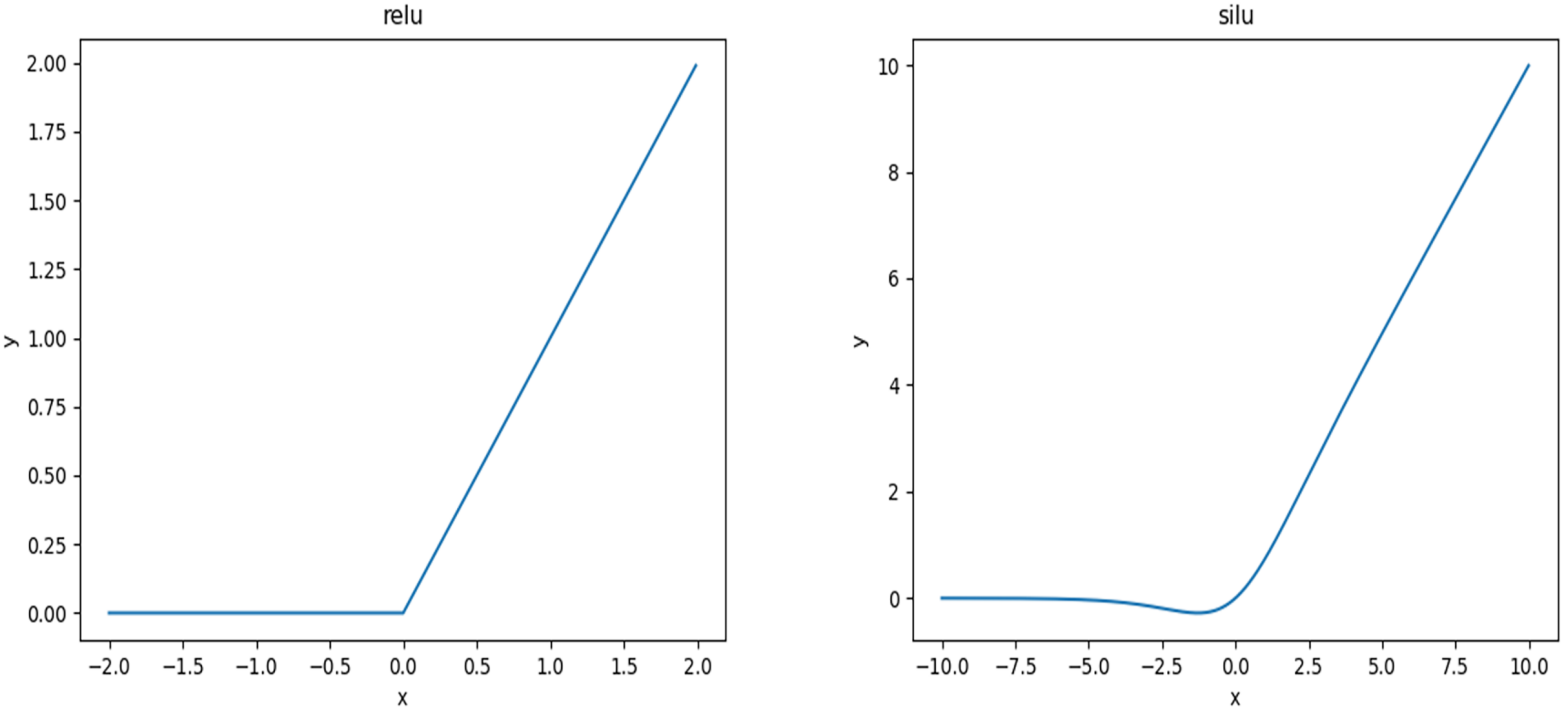
ReLU and SiLU activation functions.

ReLU and SiLU activation functions are shown in Fig. 5. The ReLU function can be regarded as a piecewise function, and its excellent computational properties can make the training of neural networks more efficient. Since the SiLU function uses the Sigmoid function, the output range of its network can be 0–1. Still, if the input data is too large or small, The SiLU function may lead to gradient explosion or disappearance. Compared with the ReLU function, the SiLU function has a smoother curve in the negative spine close to 0. In contrast, the ReLU function is relatively stable, so the activation function in the SPPF module is changed from SiLU to ReLU activation function, forming SimSPPF. After experimental verification, under the circumstances of unchanged parameter amount and calculation amount, only the activation function is changed from SiLU to ReLU function, and the running time of a convolution module is reduced by 0.427 s. Compared with the SPPF module, the operation time of the SimSPPF module is reduced by 0.860 s, the speed of convolution module increasing by 13.2% and the SPPF module by 7.2%.

### Improved YOLOv5 model

**Figure 6:**
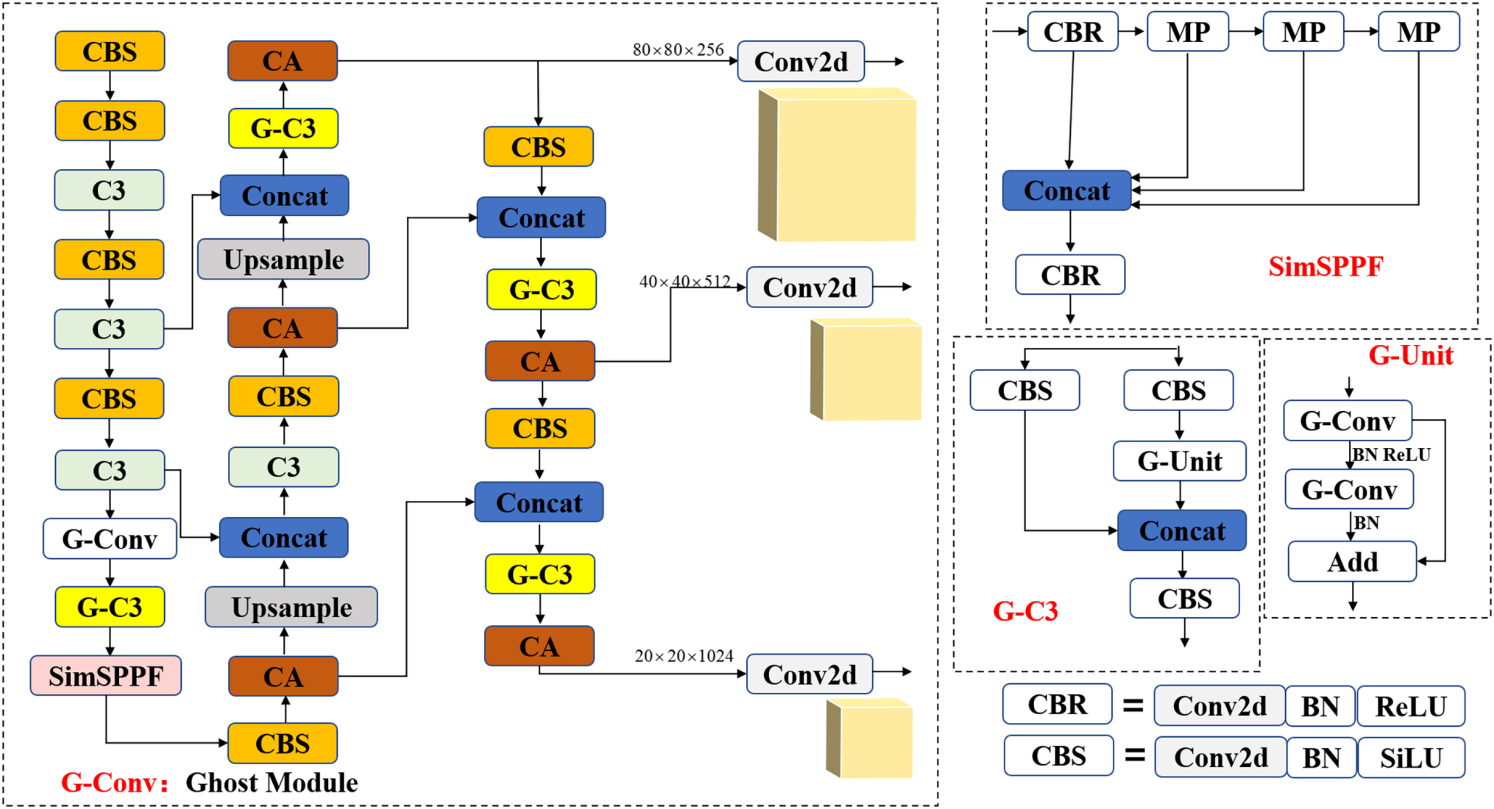
Improved YOLOv5 model.

Combining the above three improvements, including the addition of the Ghost module, CA mechanism, and modifying the SPPF module, this article proposes an optimized lightweight YOLOv5 target detection network, whose structure is shown in Fig. 6. The main improvements include: To optimize the parameter number and computational complexity of the YOLOv5 algorithm, specific conventional convolutional layers with a relatively large number of output channels are replaced with Ghost convolutions. Furthermore, Ghost Bottleneck is introduced into the C3 module to form the G-C3 module. This optimization technique aims to reduce the overall computational burden while maintaining the effectiveness of the YOLOv5 algorithm. Replacing ordinary two-dimensional convolution with Ghost convolution can reduce the number of model parameters and calculations while not increasing the depth of the network model too much; the CA mechanism is embedded into the Neck part of the model so that during the utilization of the model, focus can be put more on critical information in the input information, reduce the focus on other details, and filter out irrelevant information, to improve the accuracy and efficiency of task processing, thereby improving the overall performance of the model; the SPPF structure plays a role in the function of feature fusion. The activation function in this structure is the SiLU activation function, which is less stable than the ReLU function After verification, the SPPF module’s activation function is replaced, becoming the SimSPPF module. In terms of parameter number and calculation, with the number being the same, the detection time is reduced by 0.860 s compared with that of the original SPPF module, indicating the improvement of the overall stability of the model.

### Data source

The datasets used in this experiment are Helmet Detection (kaggle, 2020), Pascal VOC (Everingham et al., 2007), and Tomato Detection (Lars, 2020), all of which are online public datasets. Among them, Helmet Detection and Tomato Detection are single collection of datasets with 764 and 895 images, respectively, divided into 7:2:1 ratio. The Pascal VOC dataset is a collection of Pascal VOC2007 and Pascal VOC2012, with a total of 21,503 images, including 20 categories such as people, bicycles, airplanes. Due to the sizable dataset size, it is divided into an 8:1:1 ratio. On the input side of the YOLOv5s network, the input image is resized to a basic size, with the default basic size in YOLOv5s being 640 × 640 pixels. If the input image’s size is smaller than the base size, it will be resized to the basic size using an interpolation algorithm. If the input image’s size is larger than the base size, it will be resized to a smaller size and appropriately adjusted in the subsequent scaling process.

#### Experiment settings and evaluation metrics

This article conducts model training and testing with the Windows Server 2019 system. The CPU is Intel(R) Core (TM) i9-10920X CPU@3.50 GHz, the GPU is two NVIDIA GeForce RTX3090, and the video memory is 24 GB. PyTorch deep learning framework is used for model building and improvement. The Cuda version is 11.3; cudnn version is 8302; Python version is 3.8; batch_size is 64; epoch is 300; learning rate is 0.01; cosine annealing hyperparameter is 0.01; the momentum parameter in the momentum gradient descent is 0.937, and the weight decay coefficient is 0.0005.

To verify the performance of the model, the precision ( $P$) and recall ( $R$) of the test set are calculated, and the mAP, the number of model parameters, and the time required to detect a single image are used as evaluation indicators. mAP@0.5 indicates the mAP when the IOU threshold is 0.5. The calculation formula, obtained by Eqs. (4)–(6), is as follows:

(4) $$P = {T_P}/\left( {{T_P} + {F_P}} \right)$$

(5) $$R = {T_P}/\left( {{T_P} + {F_N}} \right)$$

(6) $$mAP = \displaystyle{1 \over C}\mathop \sum \nolimits_{i = 1}^C A{P_i} \times {\rm 100\%}$$

In the formula: ${T_P}$ is a positive class judged as a positive class, ${F_P}$ is a negative class considered as a positive class, ${F_N}$ is a positive class considered as a negative class, $C$ is the number of categories in the sample.

**Figure 7:**
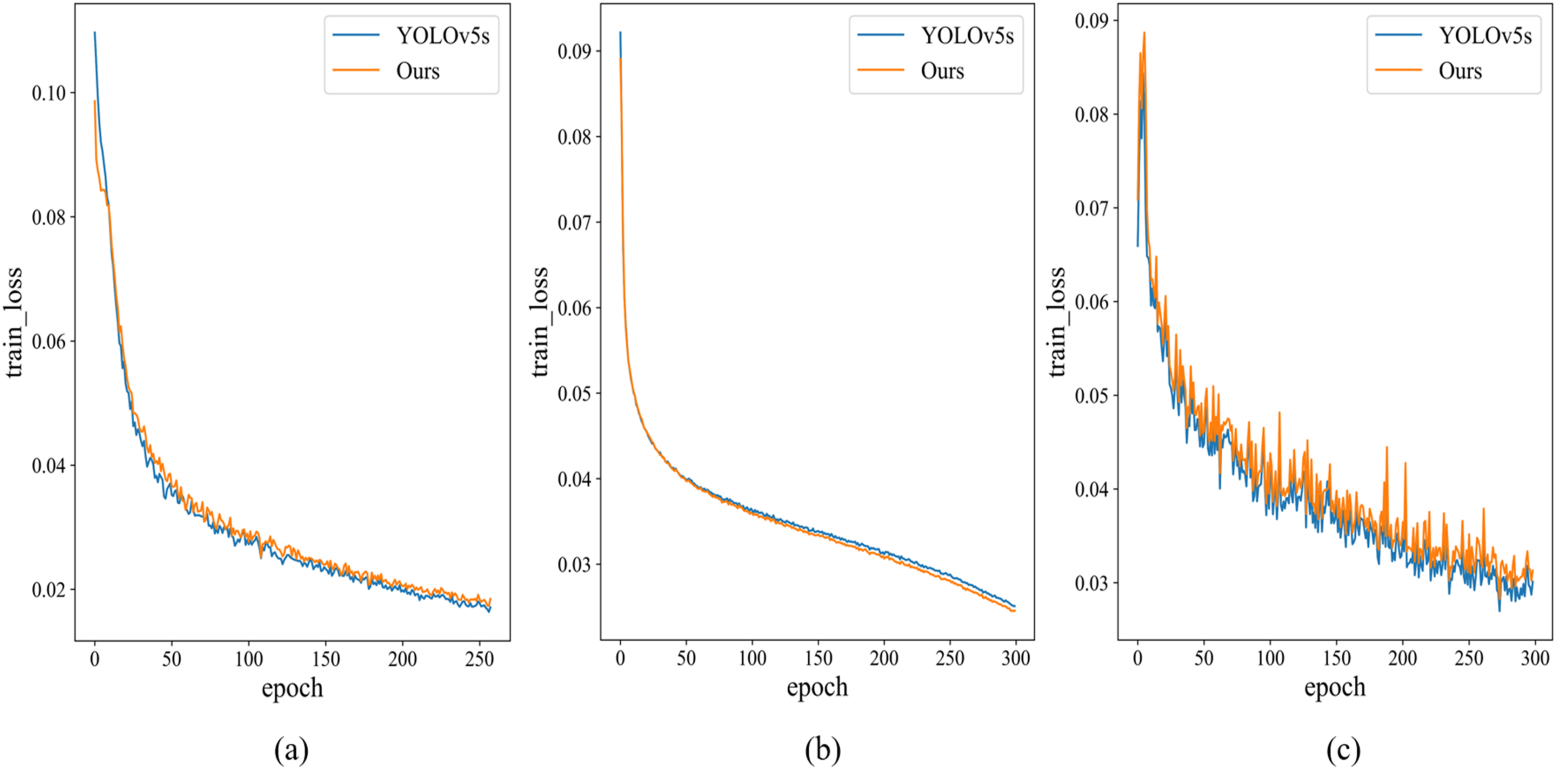
Training loss results. (A) Helmet detection dataset, (B) Pascal VOC, (C) Tomato dataset.

**Figure 8:**
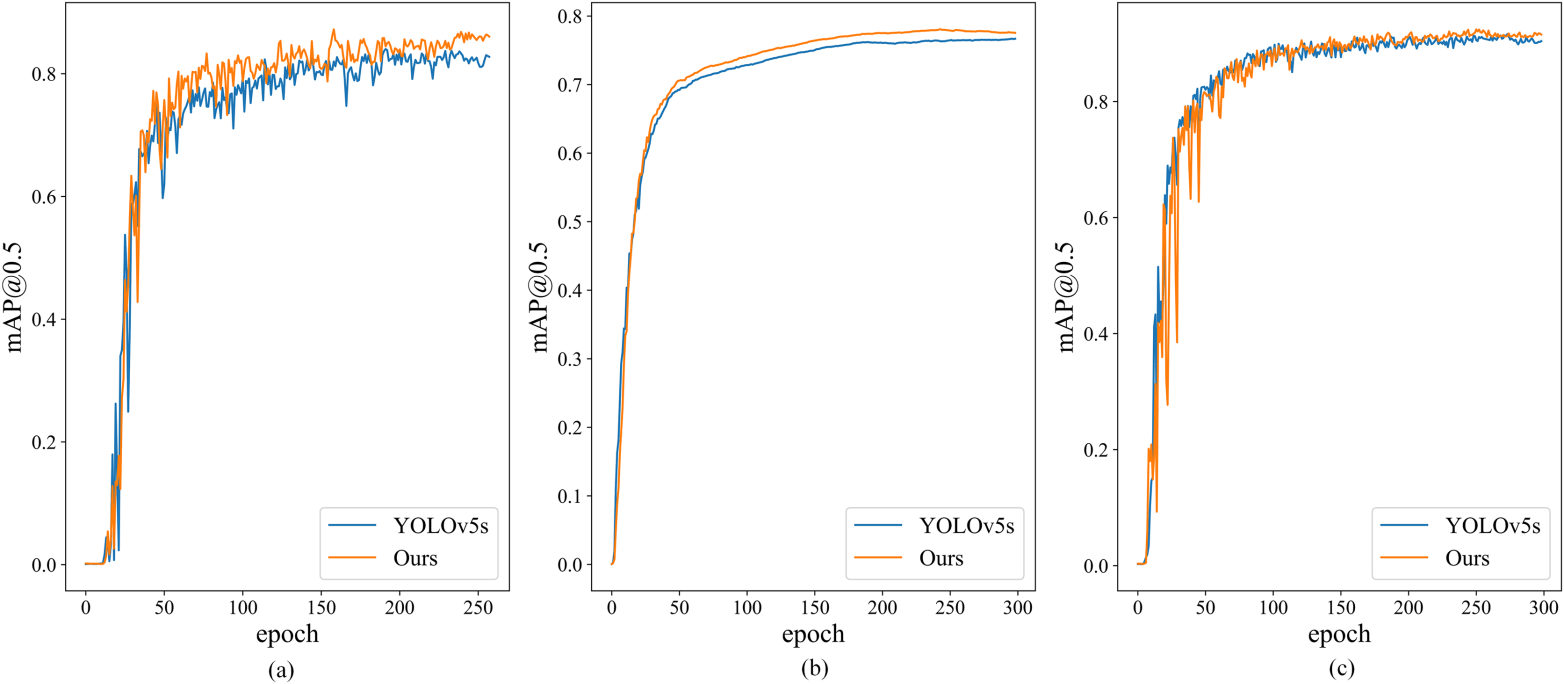
Mean average precision at IOU threshold of 0.5. (A) Helmet detection dataset, (B) Pascal VOC, (C) Tomato dataset.

The training loss functions for Helmet Detection, Pascal VOC, and Tomato Detection, as chosen in this study, are depicted in Fig. 7. Notably, the modified model exhibits rapid convergence during Helmet Detection, prompting training to cease after the 258 iterations. In contrast, YOLOv5s’ training on the Helmet Detection data continues for 300 rounds. To ensure a fair comparison, data from the 258 iterations are used, since the other two datasets undergo 300 iterations. The mAP@0.5 comparison chart is shown in Fig. 8. It is evident that, even when the training loss of the improved model surpasses that of the YOLOv5s model, mAP@0.5 still outperforms the YOLOv5s model.

The weights obtained by the model improved by us on the three datasets for 300 rounds of training are used for real-time detection on the random picture. It shows that the improved model has better detection accuracy compared with the YOLOv5 original model. There is also a better performance in the detection of object omissions.

## Experiment results and discussion

### Performance of different feature fusion modules

**Figure 9:**
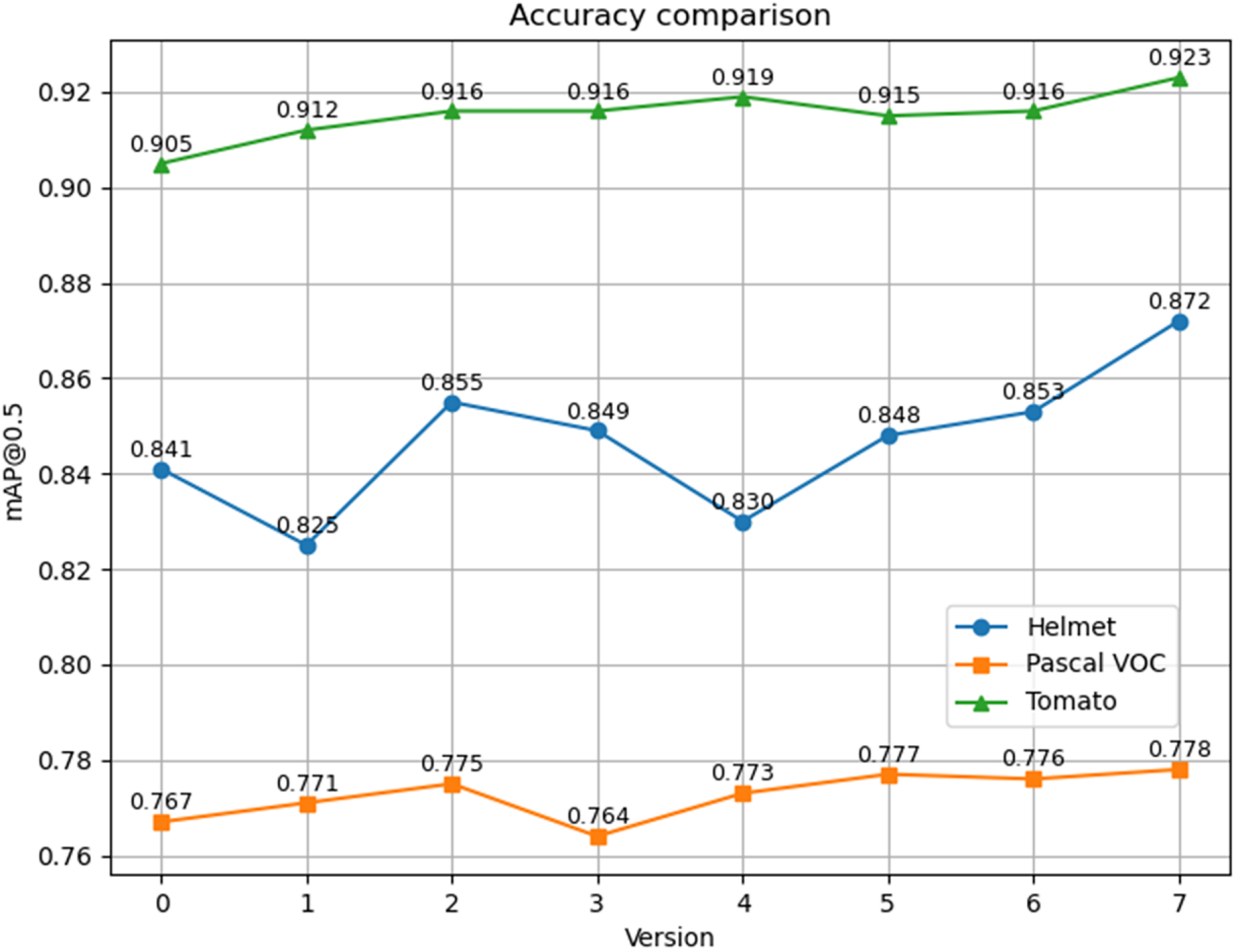
mAP of different versions on different datasets.

To evaluate the impact and efficacy of employing the Ghost module, CA mechanism and SimSPPF module, ablation experiments were conducted using YOLOv5s as the baseline. The experiments in the Helmet Detection dataset are shown in Table 2. It can be seen that the mAP@0.5 of the YOLOv5s model is 84.1%; after using the CA mechanism and the SimSPPF module alone, the accuracy is improved. After adding three modules, the mAP@0.5 is 87.2%. The Ghost module effectively reduces the parameter number by 2M. However, due to the need for CA to manage information embedding and CA, two additional steps are introduced in the encoding process. While this does lead to a slight increase in computational load, resulting in an additional 0.003 s in inference time, it also yields an improvement in accuracy. The associated computational cost is almost negligible and remains well within the requirements for real-time detection; ablation experiments on Pascal VOC As shown in Table 3, the mAP@0.5 of the YOLOv5s model is 76.7%, and the mAP@0.5 after the addition of three modules is 77.8%; ablation experiments on Tomato Detection dataset are shown in Table 4, the mAP@0.5 of the YOLOv5s model is 90.5%, and the mAP@0.5 after the addition of three modules is 92.3%. In the case of the comprehensive dataset, the following results can be drawn. After the combination, the average precision is still higher than the original model of YOLOv5, and the final combination result has a 28% reduction in the number of parameters. Still, the average detection precision on the dataset has been raised by 3.1%,1.1%, and 1.8%. The mAP of all versions across the three distinct datasets is presented in Fig. 9, with Version 0 representing the YOLOv5s original model and Version 7 representing the enhanced model. Evidently, the improved model exhibits higher detection accuracy on different datasets compared with the original model, and it also boasts a significant reduction in the number of parameters when contrasted with the original model.

**Table 2:** Performance comparison of different feature fusion modules in the helmet detection dataset.

Model	Ghost	CA	SimSPPF	mAP@0.5	Parameters/million	t/s
YOLOv5s				0.841	7.01	**0.011**
Version-1	$\sqrt{}$			0.825	**5.00**	0.012
Version-2		$\sqrt{}$		0.855	7.05	0.014
Version-3			$\sqrt{}$	0.849	7.01	**0.011**
Version-4	$\sqrt{}$	$\sqrt{}$		0.830	5.04	0.014
Version-5	$\sqrt{}$		$\sqrt{}$	0.848	**5.00**	0.012
Version-6		$\sqrt{}$	$\sqrt{}$	0.853	7.04	0.014
YOLOv5s (ours)	$\sqrt{}$	$\sqrt{}$	$\sqrt{}$	**0.872**	5.04	0.014

**Note:**

The best results are in bold.

**Table 3:** Performance comparison of different feature fusion modules in the Pascal VOC dataset.

Model	Ghost	CA	SimSPPF	mAP@0.5	Parameters/million	t/s
YOLOv5s				0.767	7.01	0.011
Version-1	$\sqrt{}$			0.771	**5.00**	**0.010**
Version-2		$\sqrt{}$		0.775	7.05	0.013
Version-3			$\sqrt{}$	0.764	7.01	**0.010**
Version-4	$\sqrt{}$	$\sqrt{}$		0.773	5.04	0.014
Version-5	$\sqrt{}$		$\sqrt{}$	0.777	**5.00**	0.012
Version-6		$\sqrt{}$	$\sqrt{}$	0.776	7.04	0.014
YOLOv5s (ours)	$\sqrt{}$	$\sqrt{}$	$\sqrt{}$	**0.778**	5.04	0.014

**Note:**

The best results are in bold.

**Table 4:** Performance comparison of different feature fusion modules in the Tomato dataset.

Model	Ghost	CA	SimSPPF	mAP@0.5	Parameters/million	t/s
YOLOv5s				0.905	7.01	0.012
Version-1	$\sqrt{}$			0.912	**5.00**	**0.011**
Version-2		$\sqrt{}$		0.916	7.05	0.015
Version-3			$\sqrt{}$	0.916	7.01	0.012
Version-4	$\sqrt{}$	$\sqrt{}$		0.919	5.04	0.015
Version-5	$\sqrt{}$		$\sqrt{}$	0.915	**5.00**	0.012
Version-6		$\sqrt{}$	$\sqrt{}$	0.916	7.04	0.014
YOLOv5s (ours)	$\sqrt{}$	$\sqrt{}$	$\sqrt{}$	**0.923**	5.04	0.014

**Note:**

The best results are in bold.

### Comparison with other object detection models

**Figure 10:**
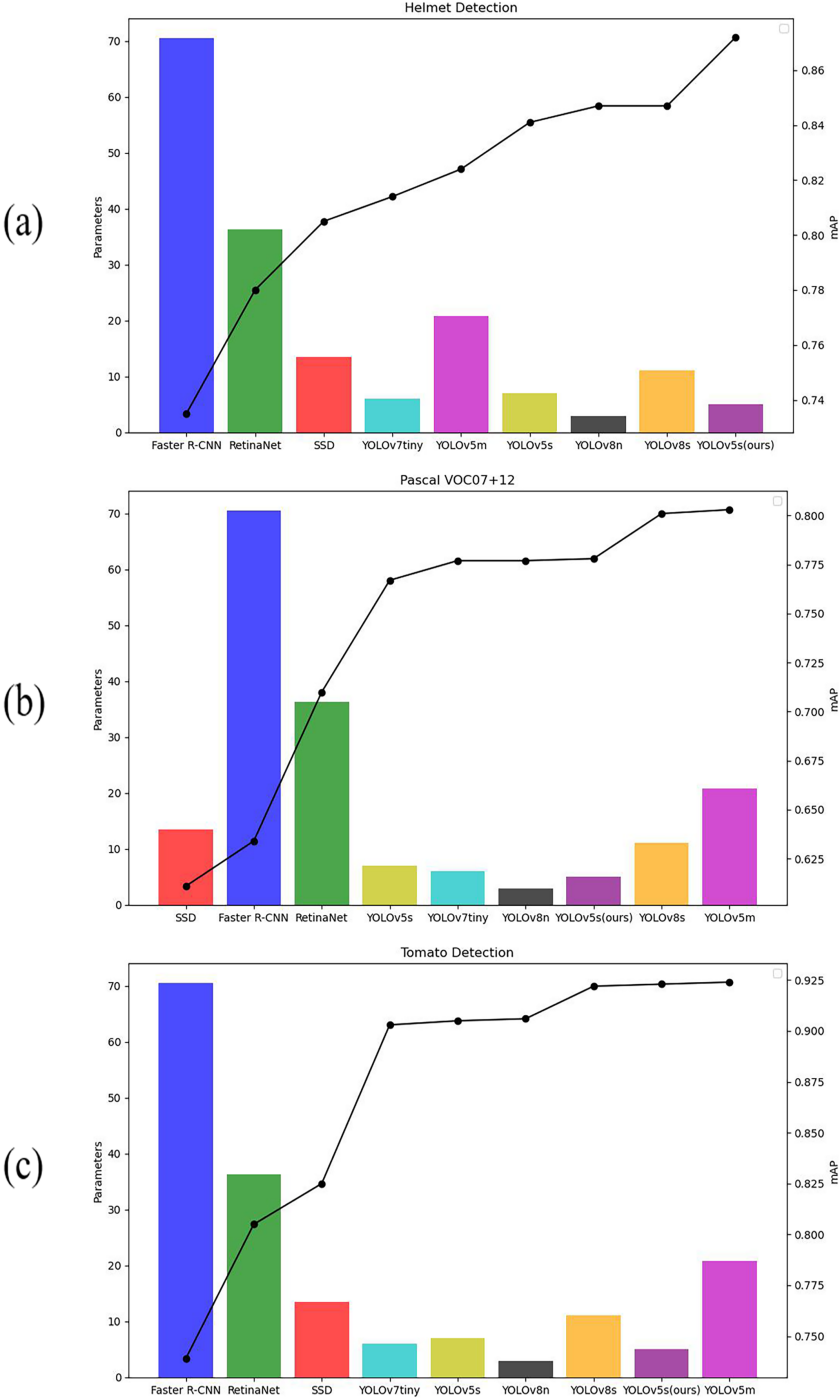
mAP and parameters of different models. (A) Helmet detection dataset, (B) Pascal VOC, (C) Tomato detection dataset.

To verify the advantages of the improved model, the three datasets, namely Helmet Detection, Pascal VOC, and Tomato Detection were compared on Faster R-CNN, YOLOv7tiny, YOLOv8s, RetinaNet, and other models. The experiment results are shown in Table 5 and the table visualization results are shown in Fig. 10. Figures 10A–10C illustrate the comparison of mAP and parameter quantities across various models on the Helmet Detection dataset, Pascal VOC dataset, and Tomato Detection dataset, respectively. The bar graph depicts the parameter quantity of each model, corresponding to the left vertical axis, while the line graph portrays the mAP values for each model, corresponding to the right vertical axis. On the Helmet Detection dataset, our improved model has achieved the highest mAP with 0.872; on both the pascal VOC dataset and the Tomato Detection dataset, the YOLOv5m algorithmic model has achieved the highest mAP, the mAP of YOLOv5m is 0.025 higher than our model on the pascal VOC dataset, and 0.001 higher than our model on the Tomato Detection dataset, but the number of parameters in YOLOv5m is four times larger than that of the model proposed in this article. When evaluating factors such as parameters, mAP, and practical deployment applications, our improved model continues to exhibit distinct advantages.

**Table 5:** The performance comparison of each algorithm (map@0.5).

Model	Helmet	Pascal VOC	Tomato	Parameters/million	t/s
YOLOv5s	0.841	0.767	0.905	7.01	**0.011**
Faster R-CNN (Resnet50)	0.735	0.634	0.739	70.55	0.046
RetinaNet (Resnet50)	0.780	0.710	0.805	36.33	0.037
YOLOv5m	0.824	**0.803**	**0.924**	20.8	0.016
YOLOv7tiny YOLOv8n	0.814 0.847	0.777 0.777	0.903 0.906	6.06 **3.01**	0.012 0.012
YOLOv8s	0.847	0.801	0.922	11.17	0.016
SSD (MobileNetV2)	0.805	0.611	0.825	13.51	0.023
YOLOv5s (ours)	**0.872**	0.778	0.923	5.04	0.014

**Note:**

The best results are in bold.

## Conclusions

Lightweight requirements are entailed in the target detection models deployed on mobile or edge devices. However, lightweight models may lead to a reduction in detection accuracy, thus affecting device performance. In order to address these problems, we propose an improved lightweight target detection model based on YOLOv5s. Firstly, Ghost Bottleneck is added to some of the C3 modules to form G-C3 modules, and some of the ordinary convolutions in Backbone are replaced with Ghost convolutions to ensure that the model effectively reduces the model parameter number and computational requirements; secondly, the model’s immunity to interference is enhanced by the addition of a CA mechanism. This mechanism reveals properties of highlighting the essential information, paying attention to the spatial location details, and strengthening the inter-channel relation in the feature information; finally, the SPPF module is modified to SimSPPF module, and this modification not only improves the model stability, but also improves the module operation efficiency. Experiment results demonstrate that the model proposed in this article can effectively reduce the number of parameters and improve detection accuracy. Compared with the original YOLOv5 model, the number of parameters only accounts for 72%, but the detection accuracy on the three datasets has increased by 3.1%, 1.1% and 1.8%. Compared with existing lightweight state-of-the-art models, such as YOLOv7tiny and YOLOv8n, our model still has the highest mAP. In summary, the lightweight improved model proposed in this article improves detection accuracy while reducing the number of parameters, and solves the problem that lightweight models may reduce detection performance. It can be deployed more efficiently on mobile or edge devices.

The current work provides an efficient method for deploying lightweight models to mobile devices, the improved models have better performance in terms of the number of parameters and detection accuracy, in the follow-up research, we will continue to work on the lightweighting of the models, for example, by performing model compression methods such as pruning and distillation as a means to optimize the inference speed of the models; additionally, while further reducing the number of model parameters, it is essential to evaluate whether the overall model performance has been significantly impacted. This process necessitates further experiments and iterations to identify the optimal trade-off point.

## Additional Information and Declarations

### Data Availability

The following information was supplied regarding data availability: The dataset of Helmet Detection is available at Zenodo: Luo Hao. (2023). The dataset of Helmet Detection [Data set]. Zenodo. https://doi.org/10.5281/zenodo.8312397. The dataset of Pascal VOC is available at Zenodo: Luo Hao. (2023). Pascal VOC [Data set]. Zenodo. https://doi.org/10.5281/zenodo.8312614. The dataset of Tomato Detection is available at Zenodo: Luo Hao. (2023). The dataset of Tomato [Data set]. Zenodo. https://doi.org/10.5281/zenodo.8312514. The correlation weights of our three datasets on different version models are available at Zenodo: Luo Hao. (2023). Correlation weights of our three datasets. Zenodo. https://doi.org/10.5281/zenodo.10070876. The code is available at Zenodo: Luo Hao. (2023). the code of my manuscript. Zenodo. https://doi.org/10.5281/zenodo.10070865.
